# Supplementary material for: Mediterranean nekton traits: distribution, relationships and significance for marine ecology monitoring and management
Source: PeerJ. 2020 Feb 14;8:e8494. doi: 10.7717/peerj.8494 (PMC7025708; doi:10.7717/peerj.8494)
Supplement: Table S1 [file peerj-08-8494-s001.pdf]

#	Trait	Definition
1	Longevity	Maximum lifespan in years (largest ever recorded for this species)
2	Age at maturity (%)	Age at maturity as a percentage of longevity (age at maturity in years divided by longevity in years)
3	Fecundity	Number of eggs or offspring produced per year by a female
4	Hermaphroditism	Existence (or prevalence) of gonochory or hermaphroditism in this species
5	Maximum length	Maximum length recorded (largest ever recorded for this species)
6	Body shape	Prevailing outline of body shape. Categories indicating varying dimension(s) in comparison to "atractoid" (flat: length and width dominant, long: length dominant, deep: depth dominant, rounded: width and depth dominant)
7	Optimal depth	Depth range of the species
8	Optimal temperature	Temperature range preference of the species
9	Habitat type	Preference for the benthic or the pelagic habitat or benthopelagic mode of habitat use
10	Distribution	Main geographic distribution of the species
11	Seabed morphology	Species affinity to specific morphological features of the habitat (soft: soft substrates like sand, silt, mud, hard: hard substrates like rock or biogenic reef, variable: heterogenous habitat or affinity to all seabed habitat types, water column: no affinity to the seabed)
12	Spawning habitat	Species preference for the benthic or pelagic habitat for spawning
13	Temperature range	Existence of wide or small tolerance range for temperature values. Either as defined in the bibliography or, if bibliography provided minimum & maximum value, a difference of over 10o C was considered as eurythermal
14	Salinity range	Existence of wide or small tolerance range for salinity values as defined from the bibliography or taking into account relevant terminology (e.g. a species occuring in marine and brackish waters was considered euryhaline)
15	Depth range	Existence of wide or small tolerance range for depth. If the bibliography provided depth range (minimun & maximum) a difference over 200 m was considered eurybathic)
16	Seasonally migratory	Occurence or not of seasonal migrations as an aspect of life cycle
17	Trophic level	Weighted quantification of diet composition as a level above primary producers (primary producers defined as level 1, primary consumers defined as level 2 and so on)
18	Diet	Characterisation of dominant food objects of the species
19	Spawning period	Dominant season of spawning
20	Feeding behaviour	Movement pattern and behaviour regarding the approach and catch of prey items (grazer: eating sessile food items or food items with disproportionally lower mobility, active predator: predator catching food items after pursuit, ambushing predator: predator catching food items with abrupt movement)
21	Sociability	Tendency to occur (or not) in groups, size of groups and level of coordination (Schooling or shoaling) with other members of the group
22	Exposure	Level of exposure of individuals with regard to the occupied microhabitat (free: fully exposed within microhabitat, permanently cryptic: species occuring half-buried, camouflaged or in a nest, temporarily cryptic: species with close affinity to environments with vegetation or rocks, but rather motile in these environments and not cryptic)
23	Mobility	Rate, pattern and permanence of movement
