# Supplementary material for: Mediterranean nekton traits: distribution, relationships and significance for marine ecology monitoring and management
Source: PeerJ. 2020 Feb 14;8:e8494. doi: 10.7717/peerj.8494 (PMC7025708; doi:10.7717/peerj.8494)
Supplement: Table S2 — Groups characterized by quantitative traits if the quantitative trait was found to differ significantly between groups (ANOVA for optimal temperature, Kruskal-Wallis for all other traits) and the group had high or low average or median in the confidence intervals or box-and-whisker plots. Groups characterized by categorical traits if more than 90% of the species of this group possess a modality (i.e., almost all or all species of this group have this modality) and if a percentage of species higher than 20% of the expected modality percentage (estimated by the overall modality frequency in all species) have this modality. [file peerj-08-8494-s002.pdf]

	Group					
	A	B	C	D	E	F
Species example	*Thunnus thynnus*	*Sarpa salpa*	*Polyprion americanus*	*Symphodus melops*	*Muraena helena*	*Squilla mantis*
QT: Found to significantly differ between groups (Optimal temperature: ANOVA, all other traits:	↑ fecuncity<br>↑ maximum length<br>↑ trophic level<br>↑ opt. temperature	↓ optimal depth<br>↓ trophic level<br>↓ opt. temperature<br>↓ longevity<br>↓ maximum length	↑ longevity<br>↑ length<br>↑ optimal depth<br>↑ trophic level<br>↓ opt. temperature	↑ fecuncity<br>↑ opt. temperature	↑ trophic level	↓ longevity<br>↓ fecundity
CT: >90% of species & 20% higher than expected	pelagic<br>migratory<br>high mobility<br>free exposure<br>atractoid body	stenobathic<br>high mobility<br>benthopelagic<br>free exposure	benthopelagic	deep-bodied	solitary<br>permanently cryptic<br>benthic<br>pelagic spawners	solitary
CT: >90% of species, but not 20% higher than	gonochoristic				gonochoristic	gonochoristic
CT: Less than 90% of species, but 20% higher than expected	living in water column<br>schooling<br>pelagic spawners<br>active predators<br>piscivore<br>euryhaline	schooling<br>atractoid body<br>grazing feeding	temperate distribution<br>piscivore<br>medium mobility<br>active predator<br>eurybathic<br>winter spawners<br>pelagic spawners	hermaphrodite<br>medium mobility<br>variable substrate<br>subtropical distribution<br>large groups<br>benthopelagic<br>benthic spawners	piscivore<br>ambushing predator<br>ambushing movement<br>stenothermal<br>stenohaline<br>eurybathic<br>rounded body	rounded body<br>low mobility<br>benthic habitat<br>zoobenthivore<br>cryptic temporarily<br>grazing feeding<br>benthic spawning<br>soft substrate

	Group					
	1	2	3	4	5	6
Species example	*Engraulis encrasicolus*	*Xiphias gladius*	*Sarpa salpa*	*Mugil cephalus*	*Spicara smaris*	*Argentina sphyraena*
QT: Found to significantly differ between groups (Optimal temperature: ANOVA, all other traits: Kruskal-Wallis)	low maximum length<br>high fecundity	high maximum length<br>high trophic level<br>high optimal temperature	low maximum length<br>high fecundity<br>shallow depth<br>low trophic level<br>high optimal temperature	high fecundity<br>shallow depth<br>low trophic level	low longevity<br>high age-at-maturity<br>low maximum length<br>shallow depth<br>low trophic level<br>high optimal temperature	
CT: >90% of species & 20% higher than expected	zooplanktivore<br>living in water column<br>subtropical<br>schools<br>pelagic habitat<br>pelagic spawning<br>migratory<br>high mobility<br>grazing feeding type<br>free exposure<br>atractoid shape	high mobility<br>free exposure<br>active predator	subtropical<br>stenothermal<br>stenobathic<br>high mobility<br>herbivore<br>grazing feeding type<br>free exposure<br>deep shape<br>benthopelagic habitat	stenobathic<br>schools<br>omnivore<br>high mobility<br>grazing feeding type<br>free exposure<br>euryhaline<br>benthopelagic habitat<br>atractoid	stenobathic<br>high mobility<br>grazing feeding type<br>low optimal temperature	pelagic spawning<br>free exposure<br>benthopelagic habitat
CT: >90% of species, but not 20% higher than expected	gonocoristic	gonocoristic		gonocoristic		
CT: Less than 90% of species, but 20% higher than expected	euryhaline	water column<br>schools<br>piscivore<br>pelagic habitat<br>pelagic spawning<br>migratory<br>euryhaline<br>atractoid body	variable seabed<br>schools<br>non-migratory<br>large groups<br>hard seabed<br>benthic spawning<br>all year spawning	soft seabed<br>migratory<br>eurythermal	zooplanktivore<br>subtropical distribution<br>stenothermal<br>schools<br>hyperbenthivore<br>hard seabed type<br>benthopelagic<br>atractoid shape	zoobenthivore<br>tropical distribution<br>summer spawning<br>stenobathic<br>schools<br>non-migratory<br>high mobility<br>eurythermal<br>atractoid shape

	Group					
	7	8	9	10	11	12
Species example	*Micromesistius poutassou*	*Merluccius merluccius*	*Hexanchus griseus*	*Diplodus sargus*	*Serranus cabrilla*	*Dentex dentex*
QT: Found to significantly differ between groups (Optimal temperature: ANOVA, all other traits: Kruskal-Wallis)	high age-at-maturity<br>low fecundity<br>deep sea		high longevity<br>high maximum length<br>deep sea<br>high trophic level			high fecundity<br>high optimal temperature
CT: >90% of species & 20% higher than expected	free exposure<br>eurybathic<br>benthopelagic habitat	pelagic spawning<br>eurythermal<br>benthopelagic habitat	solitary<br>piscivore<br>migratory<br>eurythermal	grazing feeding type	solitary<br>grazing feeding type	subtropical<br>benthopelagic habitat<br>active predator
CT: >90% of species, but not 20% higher than expected		gonocoristic	gonocoristic			
CT: Less than 90% of species, but 20% higher than expected	temperate distribution<br>stenohaline<br>solitary<br>piscivore<br>migratory<br>atractoid shape<br>all year spawning	winter spawning<br>small groups<br>deep shape<br>active predator	variable seabed<br>temperate distribution<br>long shape<br>eurybathic<br>active predator	zoobenthivore<br>variable seabed<br>subtropical distribution<br>stenobathic<br>omnivore<br>large groups<br>hermaphrodite<br>free exposure<br>deep shape	stenohaline<br>stenobathic<br>hyperbenthivore<br>hermaphrodite<br>deep shape<br>benthic spawning	stenothermal<br>solitary<br>piscivore<br>non-migratory<br>hermaphrodite<br>hard seabed<br>eurybathic<br>benthic spawning

	Group					
	13	14	15	16	17	18
Species example	*Scorpaena scrofa*	*Hippocampus guttulatus*	*Gobius niger*	*Solea solea*	*Octopus vulgaris*	*Trigla lucerna*
QT: Found to significantly differ between groups (Optimal temperature: ANOVA, all other traits: Kruskal-Wallis)	high trophic level	low longevity<br>low fecundity<br>low maximum length<br>shallow depth	low optimal temperature		high age-at-maturity<br>low fecundity	
CT: >90% of species & 20% higher than expected	solitary<br>pelagic spawning<br>cryptic-permanently<br>benthic habitat	zooplanktivore<br>summer spawning<br>stenobathic<br>solitary<br>non-migratory<br>low mobility<br>long body<br>grazing feeding type<br>eurythermal<br>euryhaline<br>cryptic-permanently<br>benthopelagic habitat<br>benthic spawning	summer spawning<br>stenobathic<br>grazing feeding type<br>eurythermal<br>euryhaline	low mobility<br>benthic habitat	subtropical<br>stenohaline<br>eurybathic<br>cryptic-temporarily<br>benthic habitat<br>active predator	stenohaline<br>solitary<br>grazing feeding type
CT: >90% of species, but not 20% higher than expected	gonocoristic	gonocoristic	gonocoristic	gonocoristic	gonocoristic	
CT: Less than 90% of species, but 20% higher than expected	stenothermal<br>stenohaline<br>rounded shape<br>piscivore<br>eurybathic<br>ambusher movement<br>ambusher feeding type	temperate distribution<br>soft seabed	zoobenthivore<br>temperate distribution<br>solitary<br>soft seabed<br>benthic habitat	subtropical distribution<br>solitary<br>soft seabed<br>grazing feeding type<br>flat shape<br>cryptic-temporarily<br>cryptic-permanently	variable seabed<br>stenothermal<br>solitary<br>rounded shape<br>piscivore<br>migratory<br>benthic spawning	zoobenthivore<br>soft seabed<br>rounded shape<br>non-migratory<br>eurybathic<br>benthic spawning
