# Supplementary figures and images for: Mediterranean nekton traits: distribution, relationships and significance for marine ecology monitoring and management
Source: PeerJ. 2020 Feb 14;8:e8494. doi: 10.7717/peerj.8494 (PMC7025708; doi:10.7717/peerj.8494)

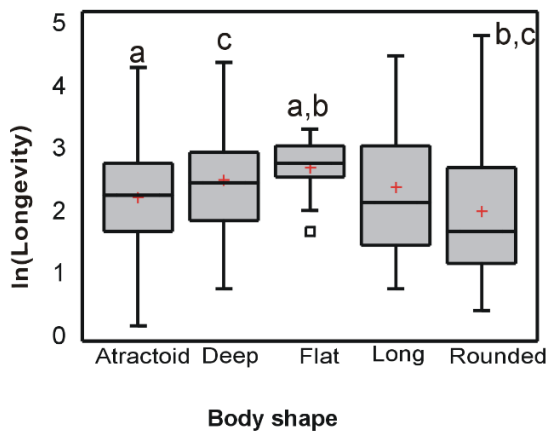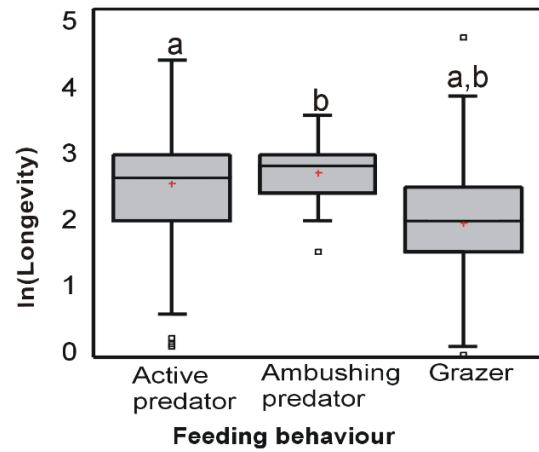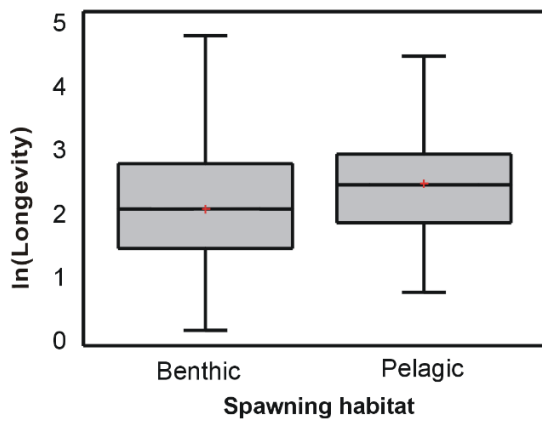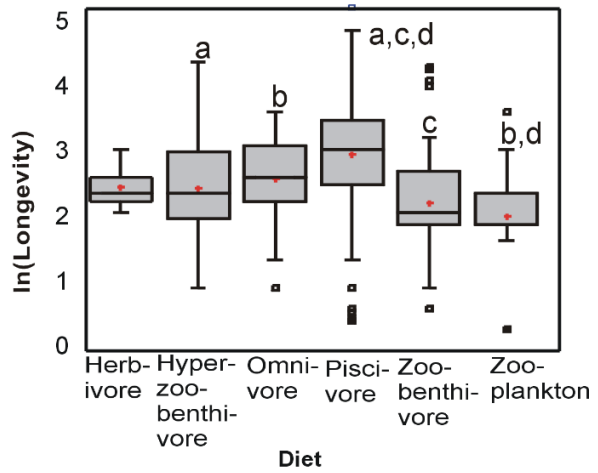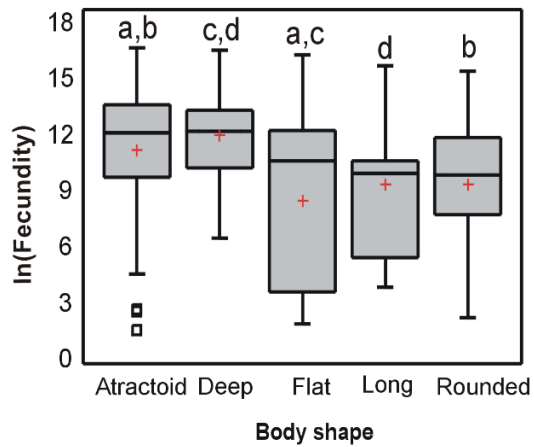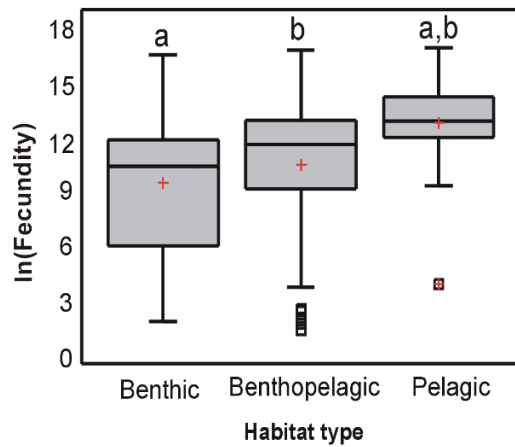

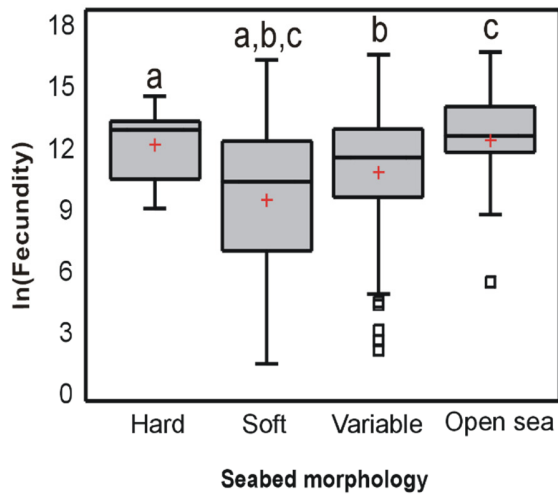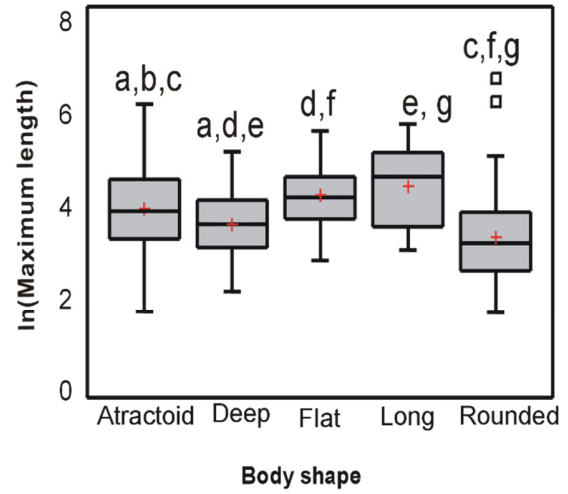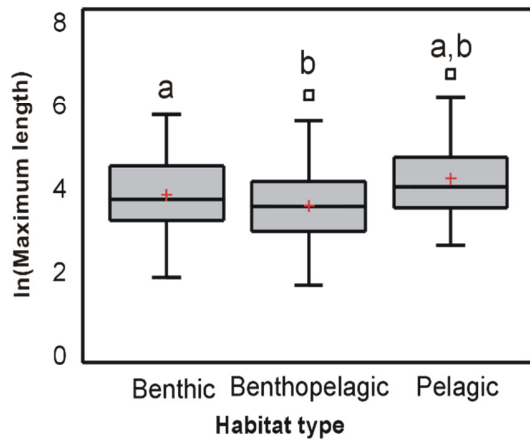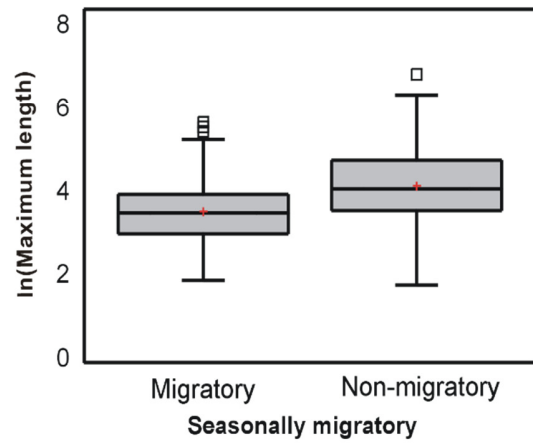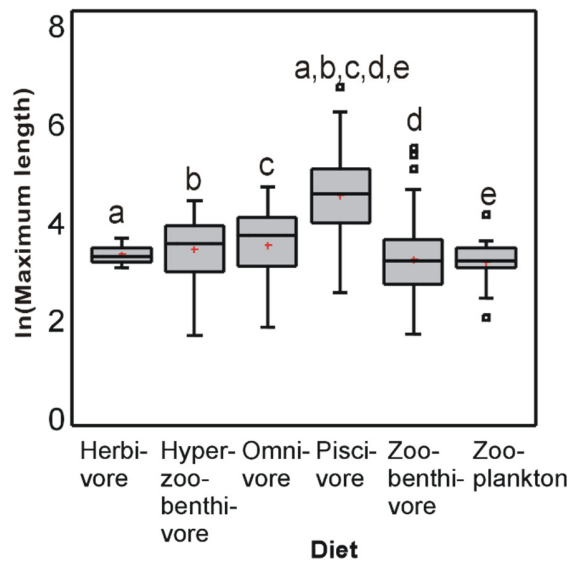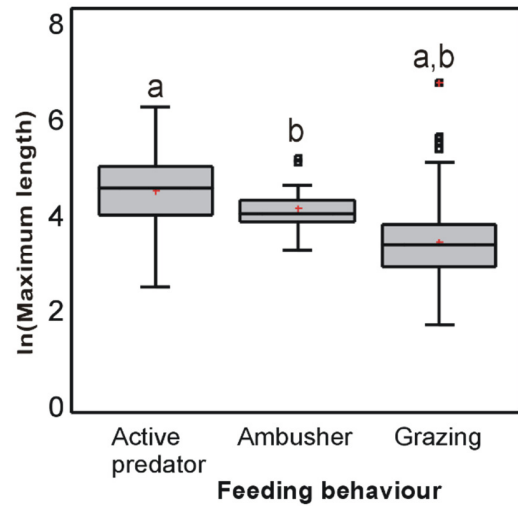

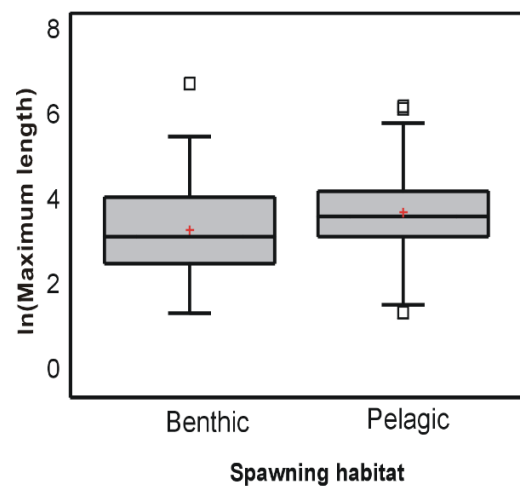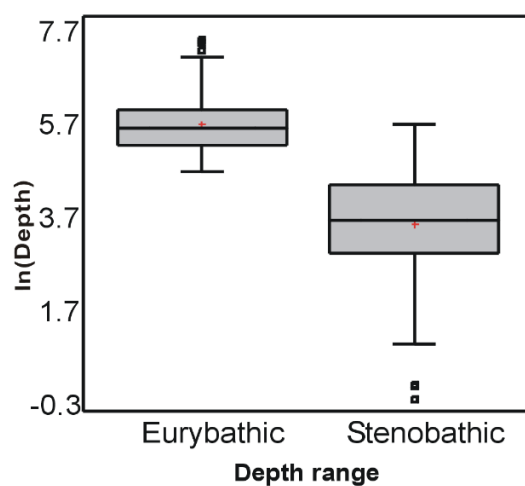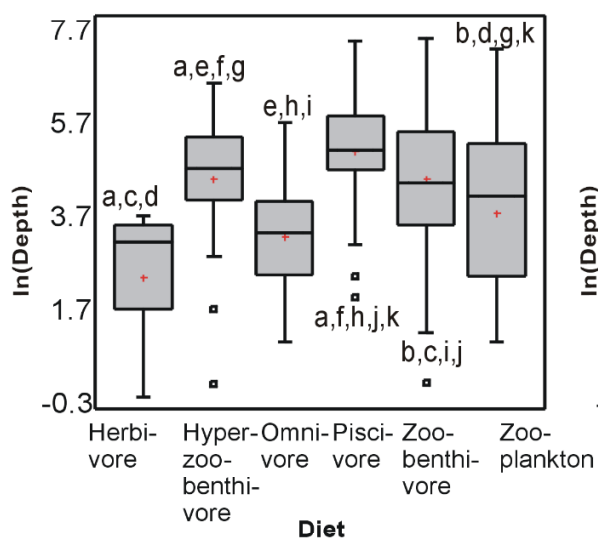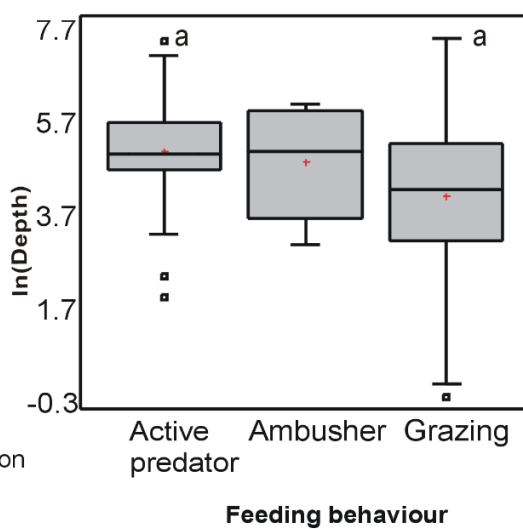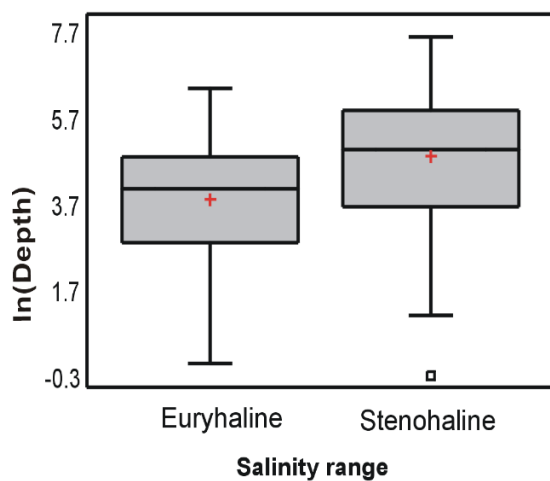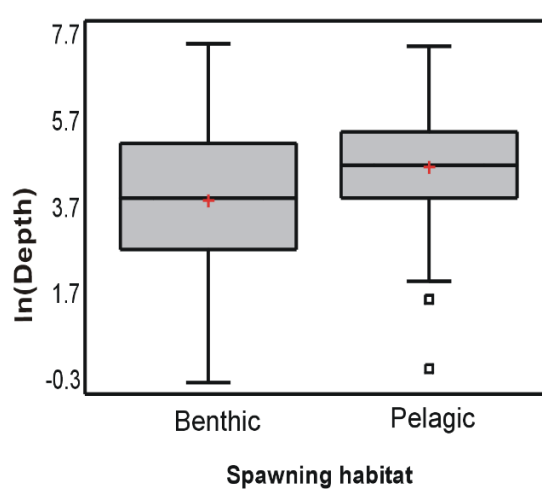

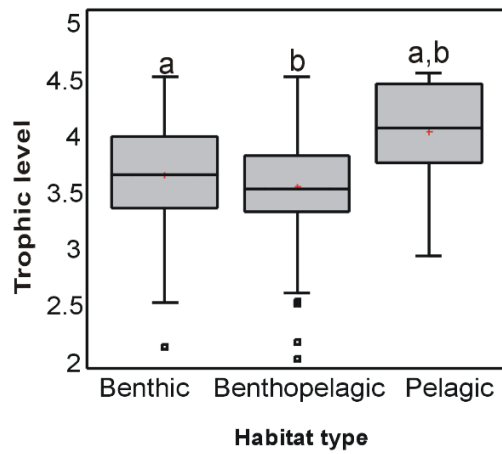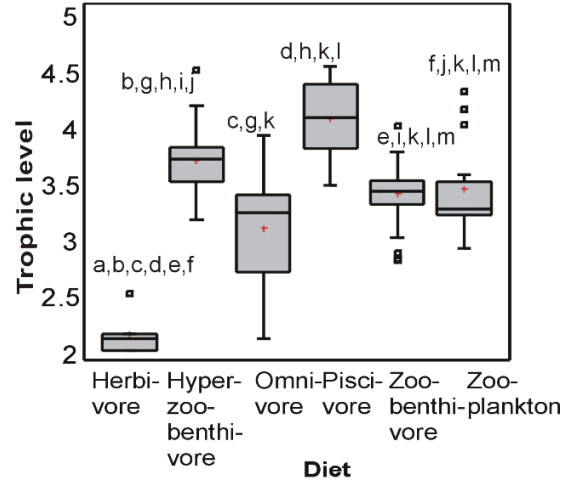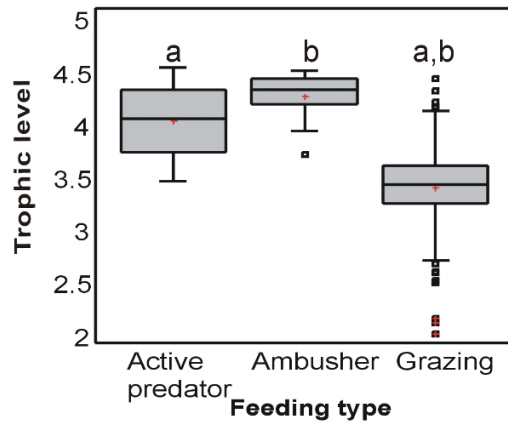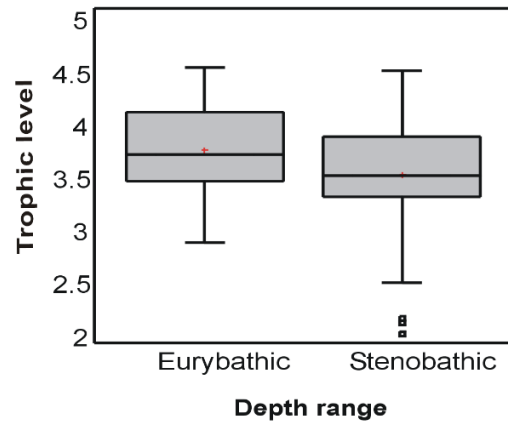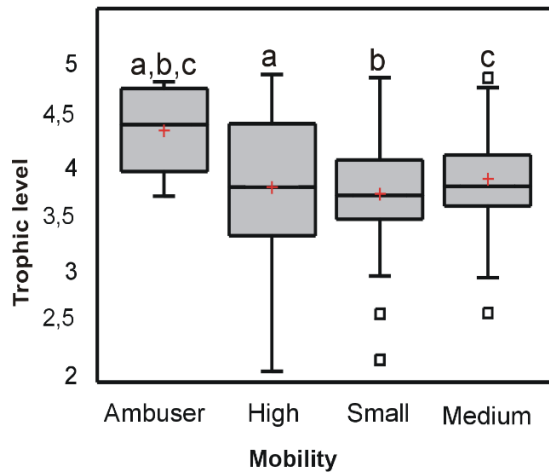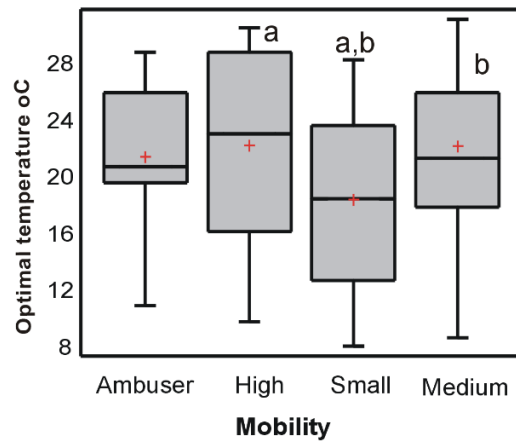

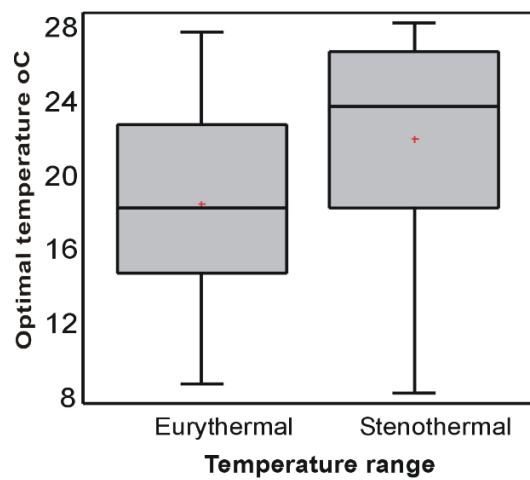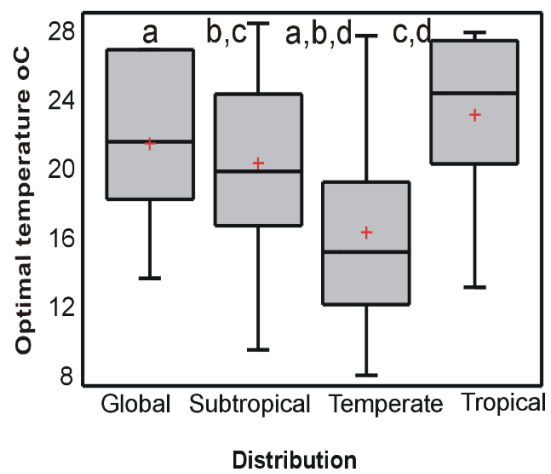

Supplement: Figure S1 — The mean value is represented with the red cross. The pairs of the same letters represent the traits categories/modalities with statistically significant differences in pairwise comparisons. [file peerj-08-8494-s003.pdf]
